# Associations Between Mother-Adolescent and Father-Adolescent Relationships and Young Adult Health

**DOI:** 10.1001/jamanetworkopen.2023.3944

**Published:** 2023-03-21

**Authors:** Carol A. Ford, Andrew C. Pool, Nicole F. Kahn, James Jaccard, Carolyn T. Halpern

**Affiliations:** 1 Division of Adolescent Medicine, Department of Pediatrics, Perelman School of Medicine at the University of Pennsylvania and Children’s Hospital of Philadelphia, Philadelphia; 2 Center for Parent and Teen Communication at Children’s Hospital of Philadelphia, Philadelphia, Pennsylvania; 3 Department of Maternal and Child Health, Gillings School of Global Public Health, University of North Carolina at Chapel Hill; 4 Silver School of Social Work at New York University, New York

## Abstract

**Importance:**

Studies linking the quality of parent-adolescent relationships with young adult health outcomes could inform investments to support these complex relationships.

**Objective:**

To evaluate whether consistently measured, modifiable characteristics of parent-adolescent relationships are associated with young adult health across multiple domains.

**Design, Setting, and Participants:**

This cohort study used data from waves I (1994-1995; ages 12-17 years) and IV (2008-2009; ages 24-32 years) of the US National Longitudinal Study of Adolescent to Adult Health. Of 20 745 adolescents enrolled in wave I, 15 701 of 19 560 who were eligible completed wave IV (response rate, 80.3%). Data analyses were conducted from February 2019 to November 2020.

**Exposures:**

Parental warmth, parent-adolescent communication, time together, relationship and communication satisfaction, academic expectations, and maternal inductive discipline as reported at wave I by adolescent participants.

**Main Outcomes and Measures:**

Wave IV participant-reported self-rated health, depression, stress, optimism, nicotine dependence, substance abuse symptoms (alcohol, cannabis, or other drugs), unintended pregnancy, romantic relationship quality, physical violence, and alcohol-related injury. Separate regression models were run for mother-adolescent and father-adolescent relationships while controlling for age, biological sex, race and ethnicity, parental educational level, family structure, and child maltreatment experiences.

**Results:**

A total of 10 744 participants (mean [SD] age at wave IV, 28.2 [1.8] years; 52.0% female; 67.3% non-Hispanic White) and 8214 participants (mean [SD] age at wave IV, 28.2 [1.8] years; 50.8% female; 71.9% non-Hispanic White) had valid sampling weights and complete data for mother-adolescent and father-adolescent relationship characteristics, respectively. Adolescents who reported higher levels of mother-adolescent warmth (β = 0.11 [95% CI, 0.06-0.15]), communication (β = 0.02 [95% CI, 0.00-0.04]), time together (β = 0.07 [95% CI, 0.05-0.09]), academic expectations (β = 0.05 [95% CI, 0.02-0.08]), relationship or communication satisfaction (β = 0.07 [95% CI, 0.04-0.10]), and inductive discipline (β = 0.03 [95% CI, 0.01-0.05]) reported significantly higher levels of self-rated general health in young adulthood. Adolescents who reported higher levels of father-adolescent warmth (β = 0.07 [95% CI, 0.03-0.11]), communication (β = 0.03 [95% CI, 0.01-0.05]), time together (β = 0.06 [95% CI, 0.03-0.08]), academic expectations (β = 0.04 [95% CI, 0.01-0.06]), and relationship satisfaction (β = 0.07 [95% CI, 0.04-0.10]) also reported significantly higher levels of self-rated general health in young adulthood. Adolescents reporting higher levels of all exposures also reported significantly higher levels of optimism and romantic relationship quality in young adulthood (β coefficient range, 0.02 [95% CI, 0.00-0.04] to 0.24 [95% CI, 0.15-0.34]) and lower levels of stress and depressive symptoms (β coefficient range, −0.07 [95% CI, –0.12 to –0.02] to −0.48 [95% CI, –0.61 to –0.35]). Higher levels of parental warmth, time together, and relationship or communication satisfaction were significantly associated with lower levels of nicotine dependence (odds ratio range, 0.78 [95% CI, 0.72-0.85] to 0.89 [95% CI, 0.81-0.98]) and substance abuse symptoms (incidence rate ratio range, 0.60 [95% CI, 0.50-0.73] to 0.94 [95% CI, 0.89-0.99]), as well as lower odds of unintended pregnancy (odds ratio range, 0.81 [95% CI, 0.74-0.88] to 0.93 [95% CI, 0.86-0.99]). Patterns were less consistent for physical violence and alcohol-related injury. Characteristics of mother-adolescent and father-adolescent relationships were similarly associated with young adult outcomes.

**Conclusions and Relevance:**

The findings of this cohort study suggest that adolescents’ positive perceptions of their relationships with their mothers and fathers are associated with a wide range of favorable outcomes in young adulthood. Investments in improving parent-adolescent relationships may have substantial benefits for young adult population health.

## Key Points

**Question:**

What characteristics of the parent-adolescent relationship are associated with young adult health outcomes?

**Findings:**

In this cohort study of more than 15 000 adolescents, higher levels of adolescent-reported parental warmth, parent-adolescent communication, time together, academic expectations, relationship and communication satisfaction, and maternal inductive discipline were associated with favorable outcomes in young adulthood.

**Meaning:**

This study suggests that investments in improving the parent-adolescent relationship may be warranted, given robust links with long-term health outcomes.

## Teaser

This cohort study uses data from the US National Longitudinal Study of Adolescent to Adult Health to evaluate whether consistently measured, modifiable characteristics of parent-adolescent relationships are associated with young adult health across multiple domains.

## Introduction

The importance of parent-adolescent relationships on the health of adult populations may influence decisions about investments to expand support for these complex relationships. Research shows that characteristics of family relationships during adolescence are associated with later health outcomes including mental health, sexual health, substance use, cardiovascular risk, and overall general health.^1,2,3,4,5,6,7,8,9,10,11,12,13^ There are, however, important limitations to existing research. First, many studies are short-term and do not use diverse samples, which limits generalizability.^14^ Second, strategies for measuring dimensions of parent-adolescent relationships vary widely, which makes it challenging to identify specific characteristics to target for intervention development.^9,15,16^ Third, most studies focus on health outcomes within selected domains, which precludes a more holistic understanding of the importance of parent-adolescent relationships on adult health across domains.^1,2,3,4,5,6,7,8,9,10,11,12,13^ Fourth, few studies include a separate focus on mother-adolescent and father-adolescent relationships, which may have important differences.^17,18^

The National Longitudinal Study of Adolescent to Adult Health (Add Health) provides an opportunity to help address these limitations by evaluating whether consistently measured, modifiable, specific characteristics of parent-adolescent relationships are associated with young adult health across multiple domains.^19^ Our primary goals were to create a clearer understanding of associations between parent-adolescent relationship characteristics and the health of adult populations and to inform future research. Previous research using an attachment theory framework has shown that the quality of parent-child relationships is associated with the health of children and adolescents.^20^ Within the context of this framework, we used Add Health to examine associations of mother-adolescent and father-adolescent relationship characteristics with young adult health in the US across the domains of general health, mental health, sexual health, substance use, and injury.

## Methods

### Overview

Add Health enrolled a large, nationally representative sample of more than 20 745 in-school adolescents who were in the 7th to 12th grades during the 1994-1995 school year (wave I).^19^ This cohort has been followed up over time. Our study focused on young adult health outcomes in the third decade of life, using data from wave IV, which was completed in 2008-2009 (n = 15 701 of 19 560 who were eligible; ages 24-32 years; response rate, 80.3%).^19^ We conducted a secondary data analysis to quantify associations between wave I adolescent-reported characteristics of mother-adolescent and father-adolescent relationships and wave IV health behaviors or outcomes. This study followed the Strengthening the Reporting of Observational Studies in Epidemiology (STROBE) reporting guideline for cohort studies.^21^ The University of North Carolina institutional review board approved all original Add Health study procedures, and participants provided written informed consent. Parental consent was required for students to participate in the study. Unless otherwise directed by the school, passive consent forms were used, such that it was assumed that a parent granted permission unless they returned a signed form indicating otherwise. However, some schools did require active consent forms in which a parent returned a signed form granting permission. The Committees for the Protection of Human Subjects at Children’s Hospital of Philadelphia approved an exemption from review for this study because data were deidentified.

### Measures

The Box summarizes our conceptual framework and measures for parent-adolescent relationship characteristics (exposure variables) and health behaviors and outcomes variables. Detailed information on the construction of measures and covariates is described in eAppendix 2 in Supplement 1.

Box. **Conceptual Framework and Measures for Parent-Adolescent Relationship Characteristics and Health Behaviors and Outcomes^a^**

**Exposure Variables: Parent-Adolescent Relationship (Wave I)**

**Warmth**

- Most of the time, your mother [father] is warm and loving toward you.
- How close do you feel to your mother [father]?
- How much do you think your mother [father] cares about you?

**Extent of Communication**

- Which of the things listed have you done with your mother [father] in the past 4 weeks?
  - Talked about someone you’re dating, or a party you went to
  - Talked about a personal problem you were having
  - Talked about your school work or grades
  - Talked about other things you’re doing in school

**Time Together**

- Which of the things listed have you done with your mother or father in the past 4 weeks?
  - Gone shopping
  - Played a sport
  - Gone to a religious service or church-related event
  - Gone to a movie, play, museum, concert, or sports event
  - Worked on a project for school

**Academic Expectations**

- How disappointed would your mother [father] be if you did not graduate from college?
- How disappointed would your mother [father] be if you did not graduate from high school?

**Relationship or Communication Satisfaction**

- Overall, you are satisfied with your relationship with your mother [father].
- You are satisfied with the way your mother [father] and you communicate with each other.

**Inductive Discipline**

- When you do something wrong that is important, your mother talks about it with you and helps you understand why it is wrong.

**Outcome Variables: Health Behaviors and Outcomes (Wave IV)**

**General Health**

- Self-rated health

**Mental Health**

- Depressive symptoms
- Stress
- Optimism

**Sexual Behavior**

- Romantic relationship quality
- Unintended pregnancy

**Substance Use**

- Smoking cigarettes
- Alcohol
- Marijuana
- Other drugs

**Injury**

- Physical violence
- Alcohol-related injury

^a^
See eAppendix 2 in Supplement 1 for more details on measures.

#### Parent-Adolescent Relationship Measures

All characteristics of parent-adolescent relationships were measured by adolescent report at wave I and are reported on a scale of 1 to 5 (where 1 indicates strongly disagree or not at all and 5 indicates strongly agree or very much). Adolescents were asked about various aspects of their relationships with their residential parent figures (ie, mother or father in the household or person who functions as mother or father in the household). Parental warmth was measured using the mean score of 3 items about love, closeness, and caring. Extent of communication was measured using the sum of 4 items on talking about social activities, personal problems, and school, then transforming the value to a scale of 1 to 5 (where 1 indicates no communication and 5 indicates communication in all categories) for ease of comparison with the other exposure measures. Time together was measured using the sum of 5 items about shared activities (eg, shopping, sports, church, events, school projects), which were transformed to a scale of 1 to 5 (where 1 indicates no activities and 5 indicates all activities). Academic expectations (graduation from high school and college) were measured using the mean score of 2 items. Adolescent relationship and communication satisfaction with parents was measured using the mean of 2 items. Each of these measures was asked separately for mother-figure and father-figure relationships. The single-scale item for inductive discipline (explaining why the adolescent has done something wrong) was asked only about mothers.

#### Health Behavior and Outcome Measures

All health behaviors and outcomes were measured by participant report at wave IV. Self-reported general health was measured with a single item on a scale of 1 to 5 (where 1 indicates poor and 5 indicates excellent). Depressive symptoms were measured using 5 items from the Center for Epidemiologic Studies Depression instrument and summing responses (range for each of the 5 items, 0-3, where 0 indicates never or rarely and 3 indicates most of the time or all of the time; total summed range for all 5 items, 0-15).^22^ Perceived stress was measured using 4 items from the Cohen Perceived Stress instrument and summing responses (range for each of the 4 items, 0-4, where 0 indicates never and 4 indicates very often for each item; total summed range for all 4 items, 0-16).^23^ Optimism was measured using 4 items on a 1 to 5 scale and summing responses (range for each of the 4 items, 1-5, where 1 indicates strongly disagree and 5 indicates strongly agree; total summed range for all items, 4-20). Romantic relationship quality was calculated as the mean score of 6 items on a scale of 1 to 5 (where 1 indicates strongly disagree and 5 indicates strongly agree) from the Supporting Healthy Marriage instrument.^24^ History of unintended pregnancy was measured with a single yes or no item. Nicotine dependence was measured using 4 items from the Heavy Smoking Index and coded as a dichotomous outcome (yes or no).^25^ Symptoms of alcohol, cannabis, and other drug abuse were based on 5-item scales from the *Diagnostic and Statistical Manual of Mental Disorders* (Fourth Edition) and summed to create count variables of the number of presenting symptoms (range, 0-4 for each, where 0 indicates no symptoms and 4 indicates all symptoms).^26^ History of physical violence and risk of alcohol-related injury were each measured with single items on scales of 0 to 3 and 0 to 2, respectively, and collapsed to create dichotomous variables (yes or no).

#### Covariates

Covariates included biological sex, race and ethnicity (Hispanic, non-Hispanic Black, non-Hispanic White, and non-Hispanic other, which included respondents of any other non-Hispanic racial group), wave I parental educational level (as a proxy for socioeconomic status), wave IV age, wave I family structure, and child maltreatment experiences reported at wave III and wave IV. These covariates were selected because they have been shown to be associated with both parent-child relationships (exposure) and health (outcome).^27,28,29^

### Statistical Analysis

Statistical analyses were conducted from February 2019 to November 2020. All statistical tests were 2-sided. Analyses were restricted to respondents who had valid sampling weights and complete data on all variables of interest. Separate models were run for mother-adolescent and father-adolescent relationships. All analyses used sampling weights and adjusted variance estimates for the Add Health complex survey design using the subpop command in Stata, version 15.9 (StataCorp LCC), to allow reporting of nationally representative estimates. The first step in the analysis was to create and measure the internal consistency of our parent-adolescent relationship domain variables. Next, regression models tested for associations between each of the parent-adolescent relationship domains and each of the outcomes while adjusting for all covariates. The Holm-Bonferroni method was used to report only statistically significant differences at the .05 level after correction for multiple tests.^30^ We used listwise deletion to address missing data. Although data were not missing completely at random (eg, they were missing primarily from single parents), the literature suggests that listwise deletion works well with large samples that have a small amount of missing data, such as Add Health, and that listwise deletion often accommodates data that are not missing at random in regression contexts, better than multiple imputation or full information maximum likelihood.^31,32,33,34^

The type of regression used depended on the strategy used for outcome measurement. Logistic regressions were used for nicotine dependence diagnosis, unintended pregnancy, physical violence, and alcohol-related injury, for which results are presented as odds ratios (ORs). Negative binomial regressions were used for alcohol abuse symptoms, cannabis abuse symptoms, and other drug abuse symptoms, for which results are presented as incidence rate ratios (IRRs). Ordinary least-squares regressions were used for depression score, stress score, optimism score, romantic relationship quality, and self-rated health, for which the β coefficients represent the estimated mean change in the outcome for every 1-unit increase in the variable. See eAppendix 1 in Supplement 1 for separate analyses focused on participants with responses to items for both the mother-adolescent and father-adolescent relationships.

## Results

### Participants

Table 1 summarizes participants with data available to investigate the association of mother-adolescent relationship characteristics with health outcomes (n = 10 744; mean [SD] age at wave IV, 28.2 [1.8] years; 52.0% female; 67.3% non-Hispanic White), and father-adolescent relationship characteristics with health outcomes (n = 8214; mean [SD] age at wave IV, 28.2 [1.8] years; 50.8% female; 71.9% non-Hispanic White). The most notable difference between samples was in family structure. Among participants reporting on mother-adolescent relationship characteristics, 20.4% were in single-parent homes, while only 3.9% of participants reporting on father-adolescent relationship characteristics were in single-parent homes.

**Table 1.** Description of Parent-Adolescent Relationship Samples and Characteristics

Characteristic	No. (%)^a^	
	Mother-adolescent relationship sample (n = 10 744)	Father-adolescent relationship sample (n = 8214)
Adolescent biological sex		
Male	4834 (48.0)	3821 (49.2)
Female	5910 (52.0)	4393 (50.8)
Adolescent race and ethnicity		
Hispanic	1623 (11.3)	1248 (11.3)
Non-Hispanic Black	2244 (14.7)	1195 (9.5)
Non-Hispanic White	5876 (67.3)	4891 (71.9)
Non-Hispanic other^b^	1001 (6.7)	880 (7.2)
Parent educational level (SES)		
<High school	1257 (11.2)	834 (9.2)
High school diploma or GED certification	2681 (27.0)	1888 (25.2)
Some college	3139 (29.9)	2377 (29.8)
College graduate	3667 (31.8)	3115 (35.7)
Age at wave IV, mean (SD), y [range, 24-32 y]	28.2 (1.8)	28.2 (1.8)
Family structure		
2 Biological parents	6191 (59.1)	6097 (76.6)
Other 2-parent family	1968 (16.8)	1552 (16.6)
Single-parent family	2292 (20.4)	327 (3.9)
Other family structure	383 (3.6)	238 (2.9)
History of child maltreatment		
No	7343 (68.8)	5797 (71.2)
Yes	3401 (31.2)	2417 (28.8)
Relationship characteristics, mean (SD) score [range, 1-5]		
Warmth	4.6 (0.6)	4.4 (0.7)
Communication	3.0 (1.3)	2.5 (1.2)
Time together	1.6 (1.1)	1.3 (1.2)
Academic expectations	4.4 (0.9)	4.4 (0.9)
Relationship or communication satisfaction	4.2 (0.9)	4.0 (1.0)
Inductive discipline	4.1 (0.9)	NA

Abbreviations: GED, General Educational Development; NA, not applicable; SES, socioeconomic status.

^a^ Percentages and means are weighted to yield national probability estimates; percentages may not sum to 100 due to rounding.

^b^ Includes respondents of any other non-Hispanic racial group.

### General Health

Adolescents who reported higher levels of mother-adolescent warmth (β = 0.11 [95% CI, 0.06-0.15]), communication (β = 0.02 [95% CI, 0.00-0.04]), time together (β = 0.07 [95% CI, 0.05-0.09]), academic expectations (β = 0.05 [95% CI, 0.02-0.08]), relationship or communication satisfaction (β = 0.07 [95% CI, 0.04-0.10]), and inductive discipline (β = 0.03 [95% CI, 0.01-0.05]) reported significantly higher levels of self-rated general health 14 years later (Table 2). Adolescents who reported higher levels of father-adolescent warmth (β = 0.07 [95% CI, 0.03-0.11]), communication (β = 0.03 [95% CI, 0.01-0.05]), time together (β = 0.06 [95% CI, 0.03-0.08]), academic expectations (β = 0.04 [95% CI, 0.01-0.06]), and relationship satisfaction (β = 0.07 [95% CI, 0.04-0.10]) also reported significantly higher levels of self-rated general health in young adulthood.

**Table 2.** Results of Ordinary Least-Squares Regression Models Testing for Associations Between Parent-Adolescent Relationship Characteristics and Adult General Health, Mental Health, and Romantic Relationship Quality

Variable	β (95% CI)				
	Self-rated health score (range, 1-5)^a^	Depression score (range, 0-15)^a^	Stress score (range, 0-16)^a^	Optimism score (range, 4-20)^a^	Romantic relationship quality score (range, 1-5)^a^
Mother-adolescent relationship (range, 1-5)^b^					
Warmth	0.11 (0.06 to 0.15)^c^	−0.33 (−0.46 to −0.21)^c^	−0.48 (−0.61 to −0.35)^c^	0.41 (0.28 to 0.55)^c^	0.15 (0.11 to 0.19)^c^
Communication	0.02 (0.00 to 0.04)^c^	−0.00 (−0.05 to 0.04)	−0.07 (−0.12 to −0.02)^c^	0.14 (0.10 to 0.18)^c^	0.02 (0.00 to 0.04)^c^
Time together	0.07 (0.05 to 0.09)^c^	−0.07 (−0.13 to −0.02)^c^	−0.18 (−0.25 to −0.11)^c^	0.19 (0.13 to 0.26)^c^	0.04 (0.02 to 0.06)^c^
Academic expectations	0.05 (0.02 to 0.08)^c^	−0.14 (−0.22 to −0.06)^c^	−0.16 (−0.26 to −0.06)^c^	0.20 (0.13 to 0.27)^c^	0.05 (0.02 to 0.08)^c^
Relationship or communication satisfaction	0.07 (0.04 to 0.10)^c^	−0.22 (−0.31 to −0.14)^c^	−0.32 (−0.41 to −0.24)^c^	0.20 (0.12 to 0.28)^c^	0.09 (0.06 to 0.11)^c^
Inductive discipline	0.03 (0.01 to 0.05)^c^	−0.12 (−0.19 to −0.04)^c^	−0.19 (−0.27 to −0.10)^c^	0.14 (0.06 to 0.21)^c^	0.07 (0.04 to 0.09)^c^
Father-adolescent relationship (range, 1-5)^b^					
Warmth	0.07 (0.03 to 0.11)^c^	−0.28 (−0.37 to −0.18)^c^	−0.38 (−0.49 to −0.26)^c^	0.24 (0.15 to 0.34)^c^	0.11 (0.08 to 0.14)^c^
Communication	0.03 (0.01 to 0.05)^c^	−0.01 (−0.07 to 0.05)	−0.08 (−0.15 to −0.01)^c^	0.11 (0.06 to 0.16)^c^	0.02 (0.00 to 0.04)^c^
Time together	0.06 (0.03 to 0.08)^c^	−0.10 (−0.16 to −0.03)^c^	−0.17 (−0.25 to −0.09)^c^	0.19 (0.13 to 0.26)^c^	0.04 (0.02 to 0.06)^c^
Academic expectations	0.04 (0.01 to 0.06)^c^	−0.10 (−0.19 to −0.01)	−0.18 (−0.28 to −0.07)^c^	0.20 (0.13 to 0.28)^c^	0.07 (0.04 to 0.10)^c^
Relationship or communication satisfaction	0.07 (0.04 to 0.10)^c^	−0.21 (−0.29 to −0.13)^c^	−0.31 (−0.40 to −0.22)^c^	0.18 (0.12 to 0.25)^c^	0.08 (0.06 to 0.11)^c^

^a^ Scores are explained in the Health Behavior and Outcome Measures subsection of the Methods section.

^b^ Scores are explained in the Parent-Adolescent Relationship Measures subsection of the Methods section.

^c^ Statistically significant at the *P* = .05 level after correcting for multiple tests using the Holm-Bonferroni method. Coefficients (β) are the estimated mean change in the outcome for every 1-unit increase in the variable, holding covariates constant. All analyses controlled for adolescent sex, age, race, ethnicity, socioeconomic status, family structure, and history of child maltreatment.

### Mental Health

Adolescents who reported higher levels of mother-adolescent warmth (β = −0.33 [95% CI, −0.46 to −0.21]), time together (β = −0.07 [95% CI, −0.13 to −0.02]), academic expectations (β = −0.14 [95% CI, −0.22 to −0.06]), relationship or communication satisfaction (β = −0.22 [95% CI, −0.31 to −0.14]), and inductive discipline (β = −0.12 [95% CI, −0.19 to −0.04]) had significantly lower levels of depressive symptoms 14 years later (Table 2). The extent of adolescent-reported mother-adolescent communication was not significantly associated with depressive symptoms in adulthood. Adolescents who reported higher levels of father-adolescent warmth (β = −0.28 [95% CI, −0.37 to −0.18]), time together (β = −0.10 [95% CI, −0.16 to −0.03]), and relationship or communication satisfaction (β = −0.21 [95% CI, −0.29 to −0.13]) had significantly lower levels of depressive symptoms in adulthood; neither academic expectations nor extent of communication with fathers was significantly associated with depressive symptoms. Adolescents who reported higher levels of mother-adolescent warmth (β = −0.48 [95% CI, −0.61 to −0.35]), communication (β = −0.07 [95% CI, −0.12 to −0.02]), time together (β = −0.18 [95% CI, −0.25 to −0.11]), academic expectations (β = −0.16 [–0.26 to −0.06]), relationship or communication satisfaction (β = −0.32 [95% CI, −0.41 to −0.24]), and inductive discipline (β = −0.19 [95% CI, −0.27 to −0.10]) reported significantly lower levels of stress in adulthood, as well as significantly higher levels of optimism (warmth: β = 0.41 [95% CI, 0.28-0.55]; communication: β = 0.14 [95% CI, 0.10-0.18]; time together: β = 0.19 [95% CI, 0.13-0.26]; academic expectations: β = 0.20 [95% CI, 0.13-0.27]; satisfaction: β = 0.20 [95% CI, 0.12-0.28]; inductive discipline: β = 0.14 [95% CI, 0.06-0.21]). Adolescents who reported higher levels of father-adolescent warmth (β = −0.38 [95% CI, −0.49 to −0.26]), communication (β = −0.08 [95% CI, −0.15 to −0.01]), time together (β = −0.17 [95% CI, −0.25 to −0.09]), academic expectations (β = −0.18 [95% CI, −0.28 to −0.07]), and relationship or communication satisfaction (β = −0.31 [95% CI, −0.40 to −0.22]) reported significantly lower levels of stress in adulthood, as well as significantly higher levels of optimism (warmth: β = 0.24 [95% CI, 0.15-0.34]; communication: β = 0.11 [95% CI, 0.06-0.16]; time together: β = 0.19 [95% CI, 0.13-0.26]; academic expectations: β = 0.20 [95% CI, 0.13-0.28]; satisfaction: β = 0.18 [95% CI, 0.12-0.25]).

### Sexual Health

Adolescents who reported higher levels of mother-adolescent warmth (β = 0.15 [95% CI, 0.11-0.19]), communication (β = 0.02 [95% CI, 0.00-0.04]), time together (β = 0.04 [95% CI, 0.02-0.06]), academic expectations (β = 0.05 [95% CI, 0.02-0.08]), relationship or communication satisfaction (β = 0.09 [95% CI, 0.06-0.11]), and inductive discipline (β = 0.07 [95% CI, 0.04-0.09]) reported significantly higher romantic relationship quality 14 years later (Table 2). Adolescents who reported higher levels of father-adolescent warmth (β = 0.11 [95% CI, 0.08-0.14]), communication (β = 0.02 [95% CI, 0.00-0.04]), time together (β = 0.04 [95% CI, 0.02-0.06]), academic expectations (β = 0.07 [95% CI, 0.04-0.10]), and relationship or communication satisfaction (β = 0.08 [95% CI, 0.06-0.11]) also reported significantly higher levels of romantic relationship quality in adulthood.

Adolescents who reported higher levels of mother-adolescent warmth (OR, 0.81 [95% CI, 0.74-0.88]), time together (OR, 0.87 [95% CI, 0.83-0.92]), and relationship or communication satisfaction (OR, 0.88 [95% CI, 0.83-0.93]) were significantly less likely to report having had an unintended pregnancy by young adulthood (Table 3). Extent of communication, academic expectations, and inductive discipline were not associated with odds of reported unintended pregnancy. Adolescents who reported higher levels of father-adolescent time together (OR, 0.91 [95% CI, 0.86-0.96]) had significantly lower odds of unintended pregnancy by young adulthood. Warmth, extent of communication, academic expectations, and relationship or communication satisfaction were not associated with odds of reported unintended pregnancy.

**Table 3.** Associations Between Parent-Adolescent Relationship Characteristics and Adult Sexual Health, Substance Use, and Injury Risk

Variable	OR (95% CI)		IRR (95% CI)			OR (95% CI)	
	Unintended pregnancy	Nicotine dependence diagnosis	Alcohol abuse symptoms	Cannabis abuse symptoms	Other drug abuse symptoms	Physical violence	Alcohol-related injury
Mother-adolescent relationship (range, 1-5)^a^							
Warmth	0.81 (0.74-0.88)^b^	0.79 (0.69-0.91)^b^	0.88 (0.80-0.96)^b^	0.74 (0.62-0.88)^b^	0.60 (0.50-0.73)^b^	0.79 (0.62-1.00)	0.86 (0.73-1.03)
Communication	0.96 (0.93-1.00)	1.04 (0.98-1.11)	1.04 (1.00-1.08)	0.98 (0.90-1.07)	0.97 (0.89-1.05)	1.04 (0.94-1.15)	1.02 (0.94-1.11)
Time together	0.87 (0.83-0.92)^b^	0.84 (0.78-0.90)^b^	0.92 (0.88-0.95)^b^	0.89 (0.79-1.00)	0.84 (0.74-0.95)^b^	1.04 (0.93-1.15)	0.87 (0.80-0.94)^b^
Academic expectations	0.96 (0.90-1.02)	0.81 (0.75-0.89)^b^	1.02 (0.94-1.12)	0.93 (0.84-1.04)	0.94 (0.82-1.07)	0.95 (0.84-1.08)	0.98 (0.84-1.14)
Relationship or communication satisfaction	0.88 (0.83-0.93)^b^	0.89 (0.81-0.98)^b^	0.88 (0.84-0.92)^b^	0.77 (0.69-0.84)^b^	0.70 (0.62-0.79)^b^	0.86 (0.75-0.99)	0.88 (0.80-0.98)
Inductive discipline	0.94 (0.89-1.00)	0.93 (0.85-1.01)	0.89 (0.85-0.94)^b^	0.90 (0.81-1.00)	0.82 (0.73-0.92)^b^	0.87 (0.74-1.01)	0.85 (0.77-0.95)^b^
Father-adolescent relationship (range, 1-5)^a^							
Warmth	0.92 (0.83-1.01)	0.80 (0.71-0.92)^b^	0.84 (0.78-0.92)^b^	0.72 (0.63-0.83)^b^	0.68 (0.58-0.81)^b^	0.70 (0.57-0.85)^b^	0.86 (0.77-0.97)
Communication	0.96 (0.92-1.01)	1.03 (0.95-1.12)	1.01 (0.96-1.05)	1.00 (0.92-1.09)	0.95 (0.84-1.08)	1.06 (0.92-1.22)	1.00 (0.93-1.08)
Time together	0.91 (0.86-0.96)^b^	0.78 (0.72-0.85)^b^	0.94 (0.89-0.99)^b^	0.90 (0.82-0.99)	0.78 (0.69-0.88)^b^	0.91 (0.79-1.04)	0.96 (0.88-1.04)
Academic expectations	0.98 (0.90-1.06)	0.84 (0.75-0.95)^b^	0.99 (0.90-1.08)	0.86 (0.77-0.97)^b^	0.84 (0.74-0.96)^b^	0.83 (0.73-0.95)^b^	0.96 (0.85-1.09)
Relationship or communication satisfaction	0.93 (0.86-0.99)	0.84 (0.77-0.92)^b^	0.87 (0.82-0.92)^b^	0.80 (0.72-0.88)^b^	0.75 (0.66-0.86)^b^	0.82 (0.71-0.96)^b^	0.90 (0.83-0.97)

Abbreviations: IRR, incidence rate ratio; OR, odds ratio.

^a^ Scores are explained in the Parent-Adolescent Relationship Measures subsection of the Methods section.

^b^ Statistically significant at the *P* = .05 level after correcting for multiple tests using the Holm-Bonferroni method. All analyses controlled for adolescent sex, age, race, ethnicity, socioeconomic status, family structure, and history of child maltreatment.

### Substance Use

Adolescents who reported higher levels of mother-adolescent warmth (OR, 0.79 [95% CI, 0.69-0.91]) and relationship or communication satisfaction (OR, 0.89 [95% CI, 0.81-0.98]) had significantly lower odds of nicotine dependence. Similarly, maternal warmth and relationship or communication satisfaction were associated with significantly lower incidence of alcohol abuse symptoms (warmth: IRR, 0.88 [95% CI, 0.80-0.96]; satisfaction: IRR, 0.88 [95% CI, 0.84-0.92]), cannabis abuse symptoms (warmth: IRR, 0.74 [95% CI, 0.62-0.88]); satisfaction: IRR, 0.77 [95% CI, 0.69-0.84]), and other drug use symptoms (warmth: IRR, 0.60 [95% CI, 0.50-0.73]; satisfaction: IRR, 0.70 [95% CI, 0.62-0.79]) 14 years later (Table 3). Adolescents who reported greater mother-adolescent time together were less likely to report nicotine dependence (OR, 0.84 [95% CI, 0.78-0.90]), alcohol abuse symptoms (IRR, 0.92 [95% CI, 0.88-0.95]), or other drug abuse symptoms (IRR, 0.84 [95% CI, 0.74-0.95]), but time together was not associated with cannabis abuse. Adolescents who reported higher levels of mother-adolescent academic expectations were less likely to report nicotine dependence (OR, 0.81 [95% CI, 0.75-0.89]), but academic expectations were not significantly associated with alcohol, cannabis, or other drug abuse symptoms. Adolescents who reported higher levels of mother-adolescent inductive discipline were less likely to report alcohol (IRR, 0.89 [95% CI, 0.85-0.94]) and other drug abuse symptoms (IRR, 0.82 [95% CI, 0.73-0.92]) in adulthood, although higher levels of mother-adolescent inductive discipline were not significantly associated with nicotine or cannabis use outcomes. Adolescent report of the extent of mother-adolescent communication was not meaningfully associated with substance use outcomes in adulthood.

Results from adolescent report of father-adolescent relationship characteristics and substance use in young adulthood were similar (Table 3). Father-adolescent warmth and relationship or communication satisfaction were significantly associated with lower odds of nicotine dependence (warmth: OR, 0.80 [95% CI, 0.71-0.92]; satisfaction: OR, 0.84 [95% CI, 0.77-0.92]), as well as lower incidence of alcohol (warmth: IRR, 0.84 [95% CI, 0.78-0.92]; satisfaction: IRR, 0.87 [95% CI, 0.82-0.92]), cannabis (warmth: IRR, 0.72 [95% CI, 0.63-0.83]; satisfaction: IRR, 0.80 [95% CI, 0.72-0.88]), and other drug abuse symptoms (warmth: IRR, 0.68 [95% CI, 0.58-0.81]; satisfaction: IRR, 0.75 [95% CI, 0.66-0.86]). Adolescents who reported greater father-adolescent time together were less likely to report nicotine dependence (OR, 0.78 [95% CI, 0.72-0.85]), as well as alcohol abuse (IRR, 0.94 [95% CI, 0.89-0.99]) and other drug abuse symptoms (IRR, 0.78 [95% CI, 0.69-0.88]); there was no significant association with cannabis abuse symptoms. Adolescents who reported higher levels of father-adolescent academic expectations were significantly less likely to report nicotine dependence (OR, 0.84 [95% CI, 0.75-0.95]), cannabis (IRR, 0.86 [95% CI, 0.77-0.97]), and other drug abuse symptoms (IRR, 0.84 [95% CI, 0.74-0.96]) in adulthood; there was no meaningful association with alcohol abuse symptoms. The extent of reported father-adolescent communication was not significantly associated with adult substance use outcomes.

### Risk of Physical Injury

No characteristics of mother-adolescent relationships were significantly associated with odds of experiencing physical violence 14 years later (Table 3). Adolescents who reported higher levels of maternal-adolescent time together (OR, 0.87 [95% CI, 0.80-0.94]) and inductive discipline (OR, 0.85 [95% CI, 0.77-0.95]) had lower odds of alcohol-related injury in young adulthood; warmth, extent of communication, academic expectations, and relationship or communication satisfaction were not associated with alcohol-related injury. Adolescents who reported higher levels of father-adolescent warmth (OR, 0.70 [95% CI, 0.57-0.85]), academic expectations (OR, 0.83 [95% CI, 0.73-0.95]), and relationship or communication satisfaction (OR, 0.82 [95% CI, 0.71-0.96]) had lower odds of physical violence in adulthood; there was no meaningful association with extent of communication or time together (Table 3). No characteristics of father-adolescent relationships were meaningfully associated with alcohol-related injury in adulthood.

### Patterns of Parent-Adolescent Relationship Characteristics and Adult Health Outcomes

A visual summary of these primary analyses displaying the associations of mother-adolescent and father-adolescent relationship characteristics with young adult health behaviors and outcomes, while controlling for all covariates, is provided in the eFigure in Supplement 1. In general, patterns show that characteristics of adolescents’ relationships with their mother and their father are similarly associated with adult outcomes. Adolescents’ perception of parental warmth had the most consistent favorable associations with adult outcomes across domains. Adolescent satisfaction with relationships or communication and amount of time spent together also had generally consistent favorable associations across domains. Maternal inductive discipline showed a generally consistent favorable association with adult health outcomes. Adolescent perceptions of parental academic expectations had less consistent associations with adult health outcomes, and extent of communication had little to no association.

A summary of the sample of participants with responses to both the mother and father relationship items (n = 7783) can be found in eTable 1 in Supplement 1. Results of the regression models that are mutually adjusted for the other parent’s corresponding relationship score can be found in eTables 2 and 3 in Supplement 1.

## Discussion

Our findings extend existing science to show that specific modifiable characteristics of parent-adolescent relationships are linked to favorable health behaviors and outcomes across multiple health domains 14 years later. Aligned with developmental science supporting the importance of quality of parenting relationships in child and adolescent health,^20^ we add to the literature suggesting links to health through the third decade of life. In a nationally representative sample, with a set of consistently defined measures of parent-adolescent relationship characteristics, we found adolescents’ perceptions of their relationships with parents were associated with general health, mental health, sexual health, and substance use in young adulthood. Adolescent perceptions of their relationships with their mothers and their fathers were similarly associated with young adult health outcomes, above and beyond associations with adolescent biological sex, race and ethnicity, socioeconomic status, family structure, and history of child maltreatment. Our results can help to inform specific strategies for improving complex parent-adolescent relationships that could be associated with young adult population health.

Patterns were consistent. Adolescents who reported warm, loving, close, caring relationships with their parents; spending more time with their parents; and high levels of satisfaction with their relationship and communication with parents reported having higher levels of general health, feelings of optimism, and quality of romantic relationships in young adulthood; they also reported lower levels of depressive symptoms, stress, nicotine dependence, and symptoms of substance abuse. We also found consistent favorable patterns associated with adolescents’ report of maternal inductive discipline. These findings are generally aligned with existing literature using less-specific measures for parent-adolescent relationship quality and narrower ranges of young adult outcomes. Steiner et al^8^ also used Add Health data to examine the association between family “connectedness” (using an averaged response provided for mother and father items) and young adult health. Their analyses found associations between adolescent perceptions of family connectedness and wave IV measures of sexual health and substance use, but not mental health. Chen and Harris,^1^ in Add Health analyses extended to wave V, found that higher family “cohesion” and lower levels of parent-child “conflict” were associated with lower risk of depressive symptoms throughout young adulthood into midlife. Variability in findings may be partially associated with differences in measurement strategies.^9^ Nonetheless, taken together, adolescents’ perception of the quality of their relationship with parents appears to have a long-lasting association with young adult health behaviors and outcomes.

There was some variability in patterns between parent-adolescent relationships and young adult outcomes. Adolescent reports of parental academic expectations were less consistently associated with young adult outcomes, highlighting that the associations with specific characteristics of parenting relationships may vary by health behavior or outcome. Even though adolescent-reported satisfaction with parent relationships and communication was consistently associated with young adult outcomes, reported extent of communication with parents was not, highlighting the complexity of behaviors such as communication, and their measurement, within parent-adolescent relationships. Patterns were more consistent for some health-related outcomes and behaviors than others. For example, the links between quality of parent-adolescent relationships and emotional health in young adulthood seemed most consistent, while links with injury and physical violence outcomes were inconsistent or weaker, highlighting that health behaviors and outcomes are likely associated with many other factors.^35,36,37,38^

Taken together, our results suggest that efforts to improve parent-adolescent relationship quality and adolescent perceptions of parent-adolescent relationships could have benefits for many (but not all) important young adult health behaviors and outcomes. Return on investment would go beyond benefits for adolescent health, reaching far into young adult populations and across a wide range of health domains. Building on existing and new research, it should be possible to identify and implement a portfolio of effective strategies to meet the needs of diverse parents and their adolescent children. Our results suggest that interventions focused specifically on influencing adolescents’ perceptions of relationship warmth, satisfaction with relationships and communication, time spent together, and inductive discipline should be prioritized. Although outreach is often to mothers, our findings suggest that interventions should include both mothers and fathers when feasible. Emerging research can inform such efforts, and more research is needed to explore innovative strategies, such as building peer support or mentoring father networks and mobile messaging interventions targeting fathers, to better engage fathers in adolescents’ health.^39,40^

Existing literature demonstrates effective strategies to influence parental expectancies and behaviors and favorably influence adolescents’ perceptions of parental trustworthiness, communication, and relationship satisfaction.^41,42,43^ Reviews of parenting interventions outside of clinic settings have shown favorable associations with the quality of parent-adolescent relationships, as well as health-related outcomes.^44,45,46,47^ Emerging literature shows that clinic-based interventions can effectively improve parenting communication and relationships as well as parent-adolescent communication about specific adolescent health-related outcomes in domains of sexual health, alcohol use, and teen driving.^48,49^ Our study supports the groundwork for a renewed interest in interventions and strategies designed to nurture and support the development of high-quality parent-adolescent relationships, above and beyond efforts targeting early childhood.^50^ Early-life factors associated with favorable quality of parent-adolescent relationships should be identified and supported, as well as strategies to “reset” low-quality relationships in late childhood and early adolescence when needed. All efforts to support parent-adolescent relationships must take into account that primary adult caregivers can look different across families.^51^ Diversity in personal values, cultural norms and expectations, family structure, and relationship dynamics need to be acknowledged and respected within the context of family relationships. There is a special need for more research on relationships between parents and sexual and gender minority youths.^52^

### Strengths and Limitations

This study has some strengths, including its large diverse sample, use of consistent specific measures for parent-adolescent relationships, and ability to test associations with a broad range of young adult health behaviors and outcomes. Analyses included adolescent relationships with both mothers and fathers.

This study also has some limitations. It precluded detailed analyses related to specific characteristics of caregivers, relationships with nonresidential parents, and more specific outcomes. In the future, more granular analyses that consider nuanced complexities of adolescent gender identity, relationships with specific types of parental figures, family structure, and specific outcomes are warranted. Measures were limited to those included on Add Health surveys. This sample is representative of in-school adolescents in the 1994-1995 school year, and findings may not be generalizable to out-of-school youth, later cohorts, or individuals in other countries. We used adolescent-reported measures in this study, which likely differ from parental reports or perceptions. Multiple factors not measured in the Add Health study or included in our analyses contribute to the complex health behaviors and health outcomes used in our study, and results should be placed within this context.

## Conclusions

This cohort study produces a clearer understanding of the associations between specific characteristics of parent-adolescent relationships and the health of young adult populations that can inform future research. Adolescents’ perceptions of their relationships with parents are associated with a wide range of favorable outcomes in young adulthood. Investments in improving adolescents’ relationships with their mothers and fathers may have substantial benefits for young adult population health.

## Notes

Supplement 1. **eAppendix 1.** Supplementary Analyses **eTable 1.** Demographic and Description of Parent-Adolescent Relationship Characteristics for the Sample With Responses for Both Parents **eTable 2.** Results of Ordinary Least-Squares Regression Models Testing for Associations Between Parent-Adolescent Relationship Characteristics and Adult General Health, Mental Health, and Romantic Relationship Quality, Mutually Adjusted for Other Parent (n=7,783) **eTable 3.** Associations Between Parent-Adolescent Relationship Characteristics and Adult Sexual Health, Substance Use, and Injury Risk, Mutually Adjusted for Other Parent (n=7,783) **eFigure.** Summary of Parent-Adolescent Relationship Characteristics on Adult Health Behaviors and Outcomes From Primary Analyses **eAppendix 2.** Supplement Methods Material
eReferences

Supplement 2.
Data Sharing Statement
