# Supplementary material for: Associations Between Mother-Adolescent and Father-Adolescent Relationships and Young Adult Health
Source: JAMA Netw Open. 2023 Mar 21;6(3):e233944. doi: 10.1001/jamanetworkopen.2023.3944 (PMC10031392; doi:10.1001/jamanetworkopen.2023.3944)
Supplement: Supplement 1. — eAppendix 1. Supplementary Analyses eTable 1. Demographic and Description of Parent-Adolescent Relationship Characteristics for the Sample With Responses for Both Parents eTable 2. Results of Ordinary Least-Squares Regression Models Testing for Associations Between Parent-Adolescent Relationship Characteristics and Adult General Health, Mental Health, and Romantic Relationship Quality, Mutually Adjusted for Other Parent (n=7,783) eTable 3. Associations Between Parent-Adolescent Relationship Characteristics and Adult Sexual Health, Substance Use, and Injury Risk, Mutually Adjusted for Other Parent (n=7,783) eFigure. Summary of Parent-Adolescent Relationship Characteristics on Adult Health Behaviors and Outcomes From Primary Analyses eAppendix 2. Supplement Methods Material eReferences [file jamanetwopen-e233944-s001.pdf]

Supplementary Online Content

Ford CA, Pool AC, Kahn NF, Jaccard J, Halpern CT. Associations between mother-adolescent and father-adolescent relationships and young adult health. *JAMA Netw Open.* 2023;6(3):e233944. doi:10.1001/jamanetworkopen.2023.3944

eAppendix 1. Supplementary Analyses

eTable 1. Demographic and Description of Parent-Adolescent Relationship Characteristics for the Sample With Responses for Both Parents

eTable 2. Results of Ordinary Least-Squares Regression Models Testing for Associations Between Parent-Adolescent Relationship Characteristics and Adult General Health, Mental Health, and Romantic Relationship Quality, Mutually Adjusted for Other Parent (n=7,783)

eTable 3. Associations Between Parent-Adolescent Relationship Characteristics and Adult Sexual Health, Substance Use, and Injury Risk, Mutually Adjusted for Other Parent (n=7,783)

eFigure. Summary of Parent-Adolescent Relationship Characteristics on Adult Health Behaviors and Outcomes From Primary Analyses

eAppendix 2. Supplement Methods Material

eReferences

This supplementary material has been provided by the authors to give readers additional information about their work.

eAppendix 1. Supplementary Analyses

These supplementary analyses present the results for the sample with data for both mothers and fathers (n=7,783). A summary of the differences between these and the results in the main manuscript, as well as a discussion of important limitations of this analysis, are provided below.

Summary of Results

Analyses were restricted to respondents who had valid sampling weights and complete data on all variables of interest for both the mother and father. eTable 1 presents the demographic characteristics of the sample (n=7,783). The mean age was 28.18 (SD=1.83), and most of the sample were identified as female (51.2%) and non-Hispanic White (72.0%).

eTables 2 and 3 present the mutually adjusted regression results for both parents. We see that many of the results that were statistically significant in the original analyses are no longer, though this is strongly affected by the more stringent criteria required when using the Holm-Bonferroni correction for multiple tests. Results suggest that multiple aspects of the relationship with the mother during adolescence may be more strongly associated with greater self-rated health and optimism scores and lower odds of unintended pregnancy in adulthood, while aspects of the relationship with the father during adolescence are more strongly associated with higher romantic relationship quality scores and lower odds of nicotine dependence and physical violence in adulthood.

Limitations

There was a large discrepancy in the sample sizes for the mother sample (n=10,744) compared to the father (n=8,214) and both parent (n=7,783) samples. Looking more closely at the sample demographics, we see that the sample that includes both parents is disproportionately White, with two biological parents, has greater parental educational attainment, and a lower proportion of respondents reporting child maltreatment (see Table 2 in the main manuscript). We also see that these sample size differences are not missing at random, as they are largely driven by a disproportionate number of respondents from single parent homes in the mother sample as compared to the father sample (20.4% vs. 3.9%, respectively). This is important, as excluding those from single-parent households, and the other social inequities that are associated with single parenting, may lead to inappropriate assumptions about these youth and families.

© 2023 Ford CA et al. *JAMA Network Open.*

eTable 1. Demographic and Description of Parent-Adolescent Relationship Characteristics for the Sample With Responses for Both Parents

	**Both Parent Sample,** n (%) **(N=7,783)**	
**Adolescent Biological Sex**		
Male	3,596 (48.8)	
Female	4,187 (51.2)	
**Adolescent Race/Ethnicity**		
Hispanic	1,194 (11.4)	
Non-Hispanic Black	1,116 (9.4)	
Non-Hispanic White	4,641 (72.0)	
Non-Hispanic other	832 (7.2)	
**Parental educational level (SES)**		
<High school	771 (8.9)	
High school diploma or GED certification	1,781 (25.3)	
Some college	2,245 (29.6)	
College graduate	2,986 (36.1)	
**Age at Wave IV** [Mean (SD); range 24-32]	28.18 (1.83)	
**Family Structure**		
2 biological parents	6,094 (80.5)	
Other 2-parent family	1,467 (16.4)	
Single-parent family	-- (<1)	
Other family structure	-- (2.9)	
**History of Child Maltreatment**		
No	5,562 (72.1)	
Yes	2,221 (27.9)	
**Relationship Characteristics** [range: 1-5; Mean (SD)]		
	**Mother**	**Father**
Warmth	4.61 (0.53)	4.39 (0.71)
Communication	2.99 (1.27)	2.44 (1.18)
Time Together	1.66 (1.09)	1.26 (1.17)
Academic Expectations	4.37 (0.86)	4.35 (0.91)
Satisfaction	4.19 (0.88)	4.03 (0.97)
Inductive Discipline	4.07 (0.92)	Not measured

Notes: Percentages and means are weighted to yield national probability estimates; Percentages may not sum to 100 due to rounding. Some sample sizes have been masked (--) to prevent deductive disclosure. The "non-Hispanic other" race category includes respondents of any other non-Hispanic racial group.

© 2023 Ford CA et al. *JAMA Network Open.*

eTable 2. Results of Ordinary Least-Squares Regression Models Testing for Associations Between Parent-Adolescent Relationship Characteristics and Adult General Health, Mental Health, and Romantic Relationship Quality, Mutually Adjusted for Other Parent (n=7,783)

	**Variable**	**Self-rated Health β (CI) (range: 1-5)**	**Depression Score β (CI) (range: 0-15)**	**Stress Score β (CI) (range: 0-16)**	**Optimism Score β (CI) (range: 4-20)**	**Romantic Relationship Quality β (CI) (range: 1-5)**
**Mother-Adolescent Relationship (range: 1-5)**	Warmth	0.09 (0.03, 0.15)*	-0.18 (-0.34, -0.01)	-0.22 (-0.40, -0.04)	0.36 (0.19, 0.54)*	0.12 (0.07, 0.17)*
	Communication	0.03 (-0.01, 0.05)	-0.01 (-0.08, 0.06)	-0.05 (-0.12, 0.02)	0.12 (0.05, 0.20)*	0.01 (-0.01, 0.04)
	Time Together	0.06 (0.03, 0.09)*	-0.01 (-0.10, 0.08)	-0.11 (-0.21, -0.02)	0.11 (0.02, 0.19)	0.01 (-0.01, 0.04)
	Academic Expectations	0.06 (0.00, 0.11)	-0.10 (-0.24, 0.05)	-0.06 (-0.24, 0.10)	0.15 (0.01, 0.29)	0.00 (-0.05, 0.05)
	Satisfaction	0.05 (0.01, 0.09)	-0.15 (-0.25, -0.04)	-0.21 (-0.32, -0.10)*	0.16 (0.06, 0.26)*	0.07 (0.04, 0.10)*
	Inductive Discipline	-	-	-	-	-
**Father-Adolescent Relationship (range: 1-5)**	Warmth	0.04 (0.00, 0.09)	-0.22 (-0.33, -0.11)*	-0.30 (-0.43, -0.17)*	0.14 (0.03, 0.24)	0.07 (0.04, 0.11)*
	Communication	0.02 (-0.02, 0.05)	-0.00 (-0.08, 0.08)	-0.04 (-0.12, 0.05)	0.04 (-0.03, 0.12)	0.02 (-0.00, 0.04)
	Time Together	0.02 (-0.00, 0.05)	-0.09 (-0.19, -0.01)	-0.11 (-0.21, -0.01)*	0.14 (0.07, 0.22)*	0.03 (0.01, 0.06)*
	Academic Expectations	0.00 (-0.05, 0.05)	-0.04 (-0.19, 0.11)	-0.13 (-0.29, 0.02)	0.09 (-0.05, 0.23)	0.07 (0.03, 0.12)*
	Satisfaction	0.05 (0.01, 0.18)*	-0.16 (-0.25, -0.07)*	-0.23 (-0.33, -0.13)*	0.12 (0.05, 0.20)*	0.05 (0.03, 0.08)*

Notes. *Statistically significant at the p = 0.05 level after correcting for multiple tests using the Holm-Bonferroni method. Coefficients (β) are the predicted mean change in the outcome for every one unit increase in the predictor, holding covariates constant. Values in parentheses are 95% confidence intervals. All analyses controlled for the corresponding relationship score for the other parent, adolescent sex, age, race, ethnicity, socioeconomic status, family structure, and history of child maltreatment.

© 2023 Ford CA et al. *JAMA Network Open.*

eTable 3. Associations Between Parent-Adolescent Relationship Characteristics and Adult Sexual Health, Substance Use, and Injury Risk, Mutually Adjusted for Other Parent (n=7,783)

		**Unintended Pregnancy OR (CI)**	**Nicotine Dependence Diagnosis OR (CI)**	**Alcohol Abuse Symptoms OR (CI)**	**Cannabis Abuse Symptoms IRR (CI)**	**Other Drug Abuse Symptoms IRR (CI)**	**Physical Violence OR (CI)**	**Alcohol-related Injury OR (CI)**
**Mother-Adolescent Relationship**	Warmth	0.84 (0.74, 0.95)*	0.89 (0.73, 1.09)	0.93 (0.83, 1.04)	0.86 (0.69, 1.06)	0.74 (0.60, 0.91)*	1.00 (0.74, 1.37)	0.87 (0.69, 1.08)
	Communication	0.98 (0.92, 1.04)	1.08 (0.99, 1.18)	1.06 (1.01, 1.12)	0.95 (0.83, 1.08)	1.03 (0.92, 1.15)	1.01 (0.86, 1.20)	1.03 (0.92, 1.15)
	Time Together	0.87 (0.81, 0.94)*	0.95 (0.84, 1.08)	0.96 (0.89, 1.03)	0.96 (0.81, 1.13)	1.02 (0.86, 1.20)	1.16 (0.98, 1.36)	0.92 (0.80, 1.04)
	Academic Expectations	0.94 (0.83, 1.06)	0.80 (0.68, 0.94)	1.02 (0.92, 1.14)	0.94 (0.77, 1.16)	0.98 (0.78, 1.24)	0.98 (0.79, 1.22)	0.90 (0.73, 1.10)
	Satisfaction	0.91 (0.85, 0.97)*	0.94 (0.83, 1.06)	0.91 (0.86, 0.97)*	0.80 (0.70, 0.91)*	0.77 (0.67, 0.88)*	1.04 (0.87, 1.24)	0.87 (0.76, 1.00)
	Inductive Discipline	-	-	-	-	-	-	-
**Father-Adolescent Relationship**	Warmth	0.95 (0.86, 1.06)	0.82 (0.71, 0.94)	0.87 (0.79, 0.95)	0.74 (0.64, 0.87)*	0.74 (0.63, 0.88)*	0.69 (0.56, 0.85)*	0.91 (0.79, 1.04)
	Communication	0.97 (0.91, 1.02)	0.97 (0.87, 1.07)	0.97 (0.91, 1.03)	1.04 (0.93, 1.16)	0.94 (0.81, 1.10)	1.07 (0.91, 1.28)	0.98 (0.89, 1.08)
	Time Together	0.97 (0.90, 1.04)	0.79 (0.70, 0.90)*	0.96 (0.89, 1.03)	0.91 (0.79, 1.04)	0.76 (0.65, 0.88)*	0.83 (0.70, 1.00)	0.95 (0.87, 1.03)
	Academic Expectations	1.01 (0.89, 1.15)	0.96 (0.81, 1.15)	0.98 (0.89, 1.09)	0.91 (0.76, 1.09)	0.84 (0.70, 1.01)	0.86 (0.68, 1.07)	1.05 (0.90, 1.22)
	Satisfaction	0.95 (0.89, 1.02)	0.86 (0.77, 0.96)	0.89 (0.83, 0.96)	0.87 (0.77, 0.99)	0.85 (0.73, 0.97)	0.76 (0.64, 0.90)*	0.95 (0.86, 1.04)

Notes. *Statistically significant at the p = 0.05 level after correcting for multiple tests using the Holm-Bonferroni method. OR = odds ratio, which resulted from logistic regression models with binary outcome variables (i.e., unintended pregnancy, nicotine dependence diagnosis, physical violence, alcohol-related injury). IRR = incidence rate ratio, which resulted from negative binomial regression models with count outcome variables (i.e., alcohol abuse symptoms, cannabis abuse symptoms, other drug abuse symptoms). Values in parentheses are 95% confidence intervals. All analyses controlled for the corresponding relationship score for the other parent adolescent sex, age, race, ethnicity, socioeconomic status, family structure, and history of child maltreatment.

© 2023 Ford CA et al. *JAMA Network Open.*

eFigure. Summary of Parent-Adolescent Relationship Characteristics on Adult Health Behaviors and Outcomes From Primary Analyses

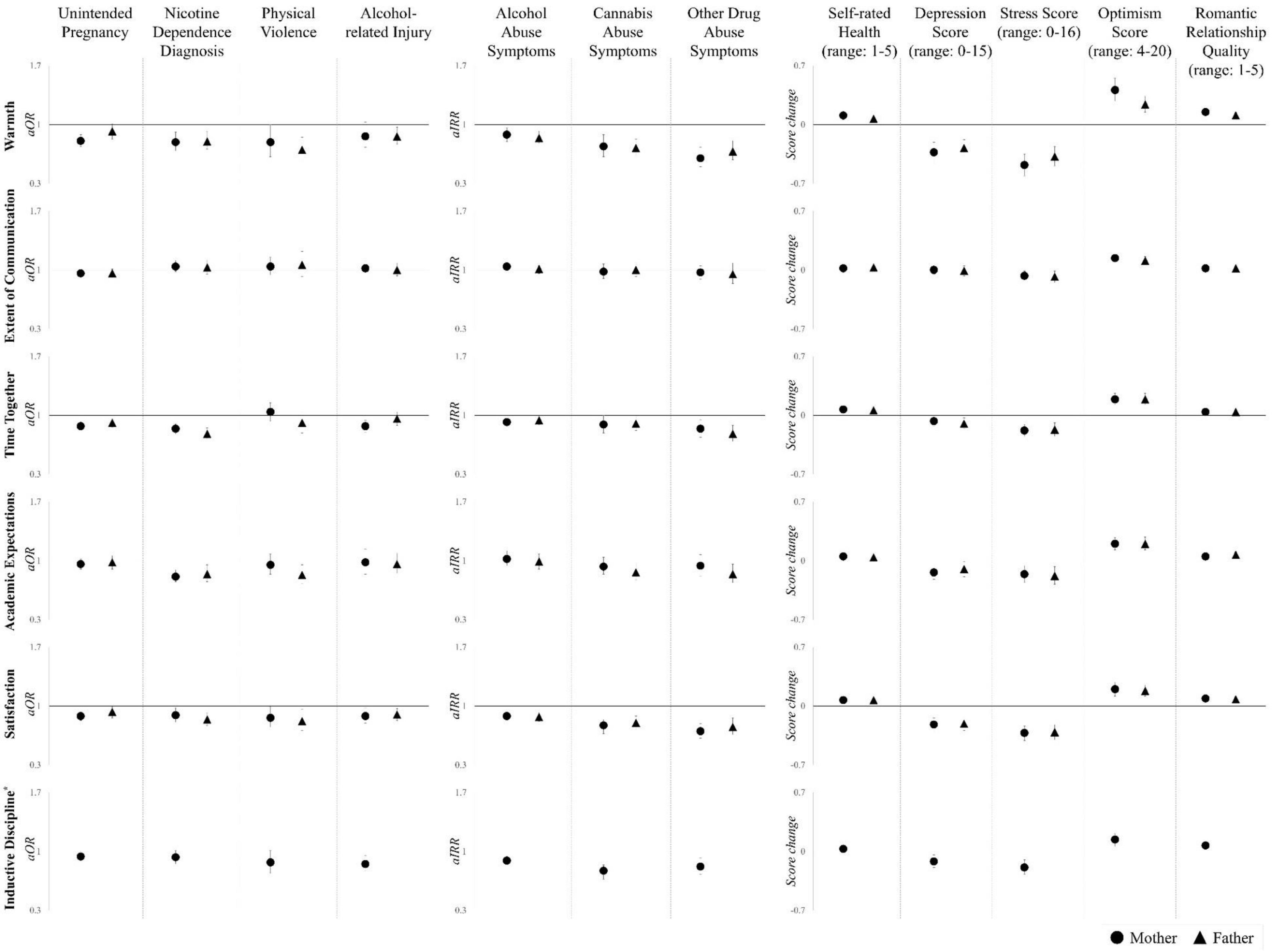

Notes. All exposure measures (labels on left) are measured on a 1-5 scale. All analyses controlled for adolescent sex, age, race, ethnicity, socioeconomic status, family structure, and history of child maltreatment. Inductive discipline only measured for mother-adolescent relationships. aOR=adjusted odds ratio; aIRR=adjusted incidence rate ratio

© 2023 Ford CA et al. *JAMA Network Open.*

eAppendix 2. Supplement Methods Material

Sample

This project uses data from four waves of the National Longitudinal Study of Adolescent to Adult Health (Add Health), a large, nationally representative sample of more than 20,745 in-school adolescents who were in the 7th-12th grades during the 1994-1995 school year (Wave I).[1] Wave II was completed one year later in 1995-1996 (n=14,738, ages 12-18; response rate=88.2%), Wave III was completed in 2001-2002 (n=15,197, ages 18-26; response rate=77.4%), and Wave IV was completed in 2008-2009 (n=15,701, ages 24-32; response rate=80.3%).[2] Our analyses are restricted to respondents who have valid sampling weights and complete data on all variables of interest.

We employed listwise deletion for missing data. Although data were not missing completely at random (e.g., they were primarily missing from single parents), the literature suggests listwise deletion works well with large samples that have a small amount of missing data, such as Add Health, and that listwise deletion often accommodates data that are not missing at random in regression contexts, better than multiple imputation or full information maximum likelihood.[3-6]

Sampling weights are used to report nationally representative estimates and are selected based on when the outcome variable(s) is measured. For these analyses, we focused on outcomes measured at Wave IV. The maximum number of respondents with valid sampling weights for Wave IV outcomes is 14,800.

Exposure Variables: Parent-Teen Relationship at Wave I

We measured the parent-teen relationship during adolescence using items from the adolescent in-home interview at Wave I, which were organized into the following six domains:

1. Parental Warmth

Adolescents were asked to rate maternal and paternal warmth, separately, using the following questions regarding warmth/love, closeness, and care:

- Most of the time, my mother/father is warm and loving toward me. (H1PF1; H1PF23)
- How close do you feel to your mother/father? (H1WP9; H1WP13)
- How much do you think she/he cares about you? (H1WP10; H1WP14)

Responses were scored from 1 = “strongly disagree” or “not at all”, to 5 = “strongly agree” or “very much,” and the mean score for each parent was calculated for analyses. The alpha coefficients were 0.71 for the mother score and 0.80 for the father score.

2. Extent of Communication

Adolescents indicated the extent of communication with their mother and father (separately) by answering the question “Which of the things listed have you done with your mother/father in the past 4 weeks?” for each the following activities:

- talked about someone you’re dating or a party you went to (H1WP17D; H1WP18D)
- talked about a personal problem you were having (H1WP17F; H1WP18F)
- talked about your school work or grades (H1WP17H; H1WP18H)
- talked about other things you’re doing in school (H1WP17J; H1WP18J)

Responses were coded as 0 = “No” and 1 = “Yes.” The communication score was the sum of all responses and ranged from 0 = “no communication” to 4 = “communication in all categories.” The alpha coefficients were 0.55 for the mother score and 0.53 for the father score. These scores were transformed to a 1-5 scale to match the range of values for the other exposure measures.

3. Time Together

© 2023 Ford CA et al. *JAMA Network Open.*

Adolescents indicated the number of shared activities spent with mother/father (assessed separately) by responding to the question “Which of the things listed have you done with your mother/father in the past 4 weeks?” with the following item choices:

- gone shopping (H1WP17A; H1WP18A)
- played a sport (H1WP17B; H1WP18B)
- gone to a religious service or church-related event (H1WP17C; H1WP18C)
- gone to a movie, play, museum, concert, or sports event (H1WP17E; H1WP18E)
- worked on a project for school (H1WP17I; H1WP18I)

Responses to these items were coded as 0 = “No” and 1 = “Yes.” The sum of the activities performed was calculated for each parent for these analyses. The alpha coefficients were 0.37 for the mother score and 0.43 for the father score.

4. Academic Expectations

Adolescents rated their mother’s/father’s educational expectations (separately) using the following items:

- On a scale of 1 to 5, where 1 is low and 5 is high, how disappointed would he/she be if you did not graduate from college? (H1WP11; H1WP15)
- On a scale of 1 to 5, where 1 is low and 5 is high, how disappointed would he/she be if you did not graduate from high school? (H1WP12; H1WP16)

As indicated in the item text, scores for each item ranged from 1 = “low” to 5 = “high.” The academic expectation score used in our analyses was the mean of these two items. The alpha coefficients were 0.85 for the mother score and 0.84 for the father score.

5. Relationship/Communication Satisfaction

The adolescent’s satisfaction regarding their relationship and communication with their mother and father was measured separately for each parent using the following items:

- Overall, you are satisfied with your relationship with your (mother/father). (H1PF5; H1PF25)
- You are satisfied with the way your (mother/father) and you communicate with each other. (H1PF4; H1PF24)

Response choices ranged from 1 = “strongly disagree,” to 5 = “strongly agree,” and the mean of the two items was used for analyses. The alpha coefficients were 0.87 for the mother score and 0.91 for the father score.

6. Inductive Discipline (mother only)

Inductive discipline was measured using adolescent responses to the following item: When you do something wrong that is important, your mother talks about it with you and helps you understand why it is wrong. (H1PF3)

Response choices ranged from 1 = “strongly disagree,” to 5 = “strongly agree.”

Outcome Variables

We measured our five outcome variables at Wave IV. The items measuring each variable are shown below.

1. General Health

<u>Self-rated Health</u>

Respondents were asked about their general health using the following item:

- In general, how is your health? (H4GH1)

Response choices ranged from 1 = “Excellent” to 5 = “poor.” Items were reverse coded so that higher scores indicated better health.

2. Mental Health

© 2023 Ford CA et al. *JAMA Network Open.*

Depression

Depressive symptoms were measured using results of the Center for Epidemiologic Studies Depression Scale (CESD).[7] This variable was already constructed using Wave IV items (C4VAR002). The items used to construct the CESD were:

- How often was the following true during the past seven days? You were bothered by things that usually don't bother you. (H4MH18)
- How often was the following true during the past seven days? You could not shake off the blues, even with help from your family and your friends. (H4MH19)
- How often was the following true during the past seven days? You had trouble keeping your mind on what you were doing. (H4MH21)
- How often was the following true during the past seven days? You were depressed. (H4MH22)
- How often was the following true during the past seven days? You were sad. (H4MH26)

Response choices to each item ranged from 0 to 3, with 0 = "Never or rarely," 1 = "Sometimes," 2 = "A lot of the time," and 3 = "Most of the time or all of the time." These numbers were converted to scores and summed to create a 0-15 scale, with higher scores indicating more depressive symptoms.

Stress

Stress was measured using the Cohen Perceived Stress Scale.[8] This variable was previously constructed using the following items from Wave IV:

- In the last 30 days, how often have you felt that you were unable to control the important things in your life? (H4MH3)
- In the last 30 days, how often have you felt confident in your ability to handle your personal problems? (H4MH4)
- In the last 30 days, how often have you felt that things were going your way? (H4MH5)
- In the last 30 days, how often have you felt that difficulties were piling up so high that you could not overcome them? (H4MH6)

Items were scored from 0 = "Never" to 4 = "Very Often." Item scores were summed to create the 0 to 16-point scale, where higher scores indicated greater stress (C4VAR002).

Optimism

The optimism scale variable was previously constructed using the following items from Wave IV:

- How much do you agree with each statement about you as you generally are now, not as you wish to be in the future? I'm always optimistic about my future. (H4PE7)
- How much do you agree with each statement about you as you generally are now, not as you wish to be in the future? I hardly ever expect things to go my way. (H4PE15)
- How much do you agree with each statement about you as you generally are now, not as you wish to be in the future? Overall, I expect more good things to happen to me than bad. (H4PE23)
- How much do you agree with each statement about you as you generally are now, not as you wish to be in the future? I rarely count on good things happening to me. (H4PE31)

Items were scored from 1 = "Strongly Disagree" to 5 = "Strongly Agree." Item scores were summed to create the 4 to 20-point scale, where higher scores indicated greater optimism (C4VAR010).

3. Sexual Behavior

Unintended Pregnancy

At Wave IV, male and female respondents provided a complete pregnancy history. For every pregnancy, respondents were asked, the following regarding fertility intentions:

- "Thinking back to the time just before this pregnancy with {fill initials}, did you want to have a child then?"

If the respondent indicated "no" for any reported pregnancy, the respondent was coded as having had an unintended pregnancy (1 = "Yes"). If none of the pregnancies was unintended or if the respondent had never been pregnant, the respondent was coded as never having an unintended pregnancy (0 = "No").

© 2023 Ford CA et al. *JAMA Network Open.*

Romantic Relationship Quality

The romantic relationship quality variable is calculated as the average score of six items from the Supporting Healthy Marriage (SHM) baseline instrument.[9] Respondents participated in this section of the Wave IV survey if they reported at least one past or present relationship with an intimate partner. Respondents were asked a series of detailed questions about their current partner, and if not currently in a relationship, their most recent partnership. Each respondent indicated how much they agreed or disagreed with the following six relationship quality items using a 5-point Likert scale (1=strongly disagree, 5= strongly agree):

- We (enjoy/enjoyed) doing even ordinary, day-to-day things together.
- I (am/was) satisfied with the way we handle our problems and disagreements;
- My partner (listens/listened) to me when I need someone to talk to;
- My partner (expresses/expressed) love and affection to me;
- I (am/was) satisfied with our sex life;
- I (trust/trusted) my partner to be faithful to me.

One item from the SHM instrument was included in the Add Health survey but was excluded from the current analysis. This item was "I (am/was) satisfied with the way we handle family finances," and is excluded because it is more applicable to marriage and cohabiting relationships than other relationship types. Items were reverse coded so that greater romantic relationship quality was indicated by a higher mean score.

4. Substance Use

Nicotine Dependence Diagnosis

A nicotine dependence diagnosis was measured using results of the Heavy Smoking Index that was constructed using items from the Wave IV interview (C4VAR018).[10] The items used to construct the Heavy Smoking Index were:

- Currently, how soon after you wake up do you have your first cigarette? (H4TO8)
- How many cigarettes a day do you smoke? (H4TO11)
- When you smoked the most, how soon after you wake up do you have your first cigarette? (H4TO15)
- When you smoked the most, how many cigarettes a day did you smoke? (H4TO18)

The Heavy Smoking Index was scored from 0 to 6. The nicotine dependence diagnosis score was coded as a dichotomous outcome, with scores of 0-3 on the Heavy Smoking Index coded as 0 = "No," and scores of 4-6 indicating 1 = "Yes."

Alcohol Abuse Symptoms

Alcohol abuse symptoms were based on the Diagnostic and Statistical Manual of Mental Disorders, Fourth Edition (DSM-IV).[11] This was a previously constructed variable based on the following items from Wave IV that ask about symptoms in the past year:

- How often has your drinking interfered with your responsibilities at work or school? (H4TO46)
- How often have you been under the influence of alcohol when you could have gotten yourself or others hurt, or put yourself or others at risk, including unprotected sex? (H4TO47)
- How often have you had legal problems because of your drinking, like being arrested for disturbing the peace or driving under the influence of alcohol, or anything else? (H4TO48)
- How often have you had problems with your family, friends, or people at work or school because of your drinking? (H4TO49)
- Did you continue to drink after you realized drinking was causing you problems with family, friends, or people at work or school? (H4TO50)

Scores were summed to create a count variable of the number of presenting symptoms, ranging from 0 to 4 (C4VAR019).

Cannabis Abuse Symptoms

Cannabis abuse symptoms were based on the Diagnostic and Statistical Manual of Mental Disorders, Fourth Edition (DSM-IV).[11] This was a previously constructed variable based on the following items from Wave IV that ask about symptoms in the past year:

© 2023 Ford CA et al. *JAMA Network Open.*

- How often has your marijuana use interfered with your responsibilities at work or school? (H4TO76)
- How often have you been under the influence of marijuana when you could have gotten yourself or others hurt, or put yourself or others at risk, including unprotected sex? (H4TO77)
- How often have you had legal problems because of your marijuana use, like being arrested for disturbing the peace or anything else? (H4TO78)
- How often have you had problems with your family, friends, or people at work or school because of your marijuana use? (H4TO79)
- Did you continue to use marijuana after you realized using it was causing you problems with family, friends, or people at work or school? (H4TO80)

Scores were summed to create a count variable of the number of presenting symptoms, ranging from 0 to 4 (C4VAR024).

Other Drug Abuse Symptoms
Other drug abuse symptoms were based on the Diagnostic and Statistical Manual of Mental Disorders, Fourth Edition (DSM-IV).[11] This was a previously constructed variable based on the following items from Wave IV that ask about symptoms in the past year:

- How often has your {favorite drug} use interfered with your responsibilities at work or school? (H4TO104)
- How often have you been under the influence of {favorite drug} when you could have gotten yourself or others hurt, or put yourself or others at risk, including unprotected sex? (H4TO105)
- How often have you had legal problems because of your {favorite drug} use, like being arrested for disturbing the peace or anything else? (H4TO106)
- How often have you had problems with your family, friends, or people at work or school because of your {favorite drug} use? (H4TO107)
- Did you continue to use {favorite drug} after you realized using it was causing you problems with family, friends, or people at work or school? (H4TO108)

Scores were summed to create a count variable of the number of presenting symptoms, ranging from 0 to 4 (C4VAR029).

5. Injury

Physical Violence
Respondents were asked to following item regarding the frequency of physical violence in the past year at Wave IV, only:

- In the past 12 months, how often did you get into a serious physical fight? (H4DS11)

Responses ranged from 0 to 3, with 0 = “Never,” 1 = “1 or 2 times,” 2 = “3 or 4 times,” and 3 = “5 or more times.” Since a response greater than 1 was rare, these were collapsed to create a dichotomous variable, in which 0=”Never” and 1=”Once or more.”

Alcohol-related Injury
Risk of injury associated with alcohol use was assessed using the following item:

- How often have you had legal problems because of your drinking, like being arrested for disturbing the peace or driving under the influence of alcohol, or anything else? (H4TO48)

Responses include 0 = “Never,” 1 = “One time,” and 2 = “More than one time.” Since a response greater than 1 was rare, these responses were collapsed to create the final binary variable, in which 0=”Never” and 1=”Once or more.”

Covariates

Covariates were selected based on existing literature that demonstrates relationships with parent-teen relationships or outcome measures.[12-16] The following covariates were used in all models:

- Biological sex of the adolescent at Wave I was taken from school records and confirmed by the interviewer.

© 2023 Ford CA et al. *JAMA Network Open.*

- Race and ethnicity of the adolescent was self-reported and verified by the interviewer (Hispanic, non-Hispanic [NH] Black, NH White, NH other)
- Socioeconomic status (SES) of the adolescent at Wave I was measured using the highest educational level achieved by either parent. The resulting categorical variable has four levels (<high school, high school diploma or GED certification, some college, college graduate).
- Age at the time of the Wave IV interview was calculated by finding the difference between the Wave IV interview dates and birth dates for each respondent.
- Family structure of the adolescent at Wave I is a constructed variable with four categories indicating which parents are in the household (2 biological parents, other 2-parent family, single-parent family, other family structure).
- Child maltreatment is a constructed, dichotomous measure (yes, no) indicating whether the respondent experienced childhood abuse or neglect before age 18. The variables used to derive this measure were reported retrospectively at Waves III and IV and include:
    - "How often did a parent or adult caregiver hit you with a fist, kick you, or throw you down on the floor, into a wall, or down stairs?" (H4MA3)
    - "How often had one of your parents or other adult care-givers touched you in a sexual way, forced you to touch him or her in a sexual way, or forced you to have sexual relations?" (H3MA4; H4MA5)
    - "How often had your parents or other adult caregivers not taken care of your basic needs, such as keeping you clean, or providing food or clothing?" (H3MA2)

© 2023 Ford CA et al. *JAMA Network Open.*

eReferences

1. Harris KM, Halpern CT, Whitsel E, et al. Add Health Research Design. 2009. http://www.cpc.unc.edu/projects/addhealth/design.
2. Harris KM, Udry JR, Bearman PS. *The Add Health Study: Design and Accomplishments*. Chapel Hill, NC; 2013.
3. Basilevsky A, Sabourin D, Hum D, Anderson A. Missing data estimators in the general linear model: An evaluation of simulated data as an experimental design. Communications in Statistics-Simulation and Computation. 1985 Jan 1;14(2):371-94.
4. Kim JO, Curry J. The treatment of missing data in multivariate analysis. Sociological Methods & Research. 1977 Nov;6(2):215-40.
5. Allison P. Listwise Deletion: It's NOT Evil. Statistical Horizons. Published June 13, 2014. Accessed January 31, 2023. https://statisticalhorizons.com/listwise-deletion-its-not-evil/
6. Pepinsky TB. A Note on Listwise Deletion versus Multiple Imputation. *Polit Anal*. 2018;26(4):480-488. doi:10.1017/pan.2018.18
7. Radloff LS. The CES-D scale: A self-report depression scale for research in the general population. *Appl Psychol Meas*. 1977;1(3):385-401. doi:10.1177/014662167700100306.
8. Cohen S, Kamarck T, Mermelstein R. A global measure of perceived stress. *J Health Soc Behav*. 1983;24:385-396. doi:10.2307/2136404.
9. Hsueh J, Knox V. Supporting Healthy Marriage evaluation: Eight sites within the United States, 2003-2013. 2014. doi:http://doi.org/10.3886/ICPSR34420.v2.
10. Heatherton TF, Kozlowski LT, Frecker RC, Fagerstrom KO. The Fagerström test for nicotine dependence: a revision of the Fagerstrom Tolerance Questionnaire. *Br J Addict*. 1991;86(9):1119-27.
11. American Psychiatric Association. *Diagnostic and Statistical Manual of Mental Disorders, Fourth Edition (DSM-IV)*. 4th ed. Washington, D.C.: American Psychiatric Association; 1994.
12. Lushin V, Jaccard J, Kaploun V. Parental monitoring, adolescent dishonesty and underage drinking: A nationally representative study. *J Adolesc*. 2017;57:99-107.
13. Johnson MD, Galambos NL. Paths to intimate relationship quality from parent–adolescent relations and mental health. *J Marriage Fam*. 2014;76(1):145-60.
14. Johnson MD. Parent–child relationship quality directly and indirectly influences hooking up behavior reported in young adulthood through alcohol use in adolescence. *Arch Sex Behav.* 2013;42(8):1463-72.
15. Shin SH, Edwards EM, Heeren T. Child abuse and neglect: relations to adolescent binge drinking in the national longitudinal study of Adolescent Health (AddHealth) Study. *Addict Behav*. 2009;34(3):277-80.
16. Brown SL. Family structure transitions and adolescent well-being. *Demography*. 2006;43(3):447-61.

© 2023 Ford CA et al. *JAMA Network Open.*
