# Supplementary material for: Associations Between Mother-Adolescent and Father-Adolescent Relationships and Young Adult Health
Source: JAMA Netw Open. 2023 Mar 21;6(3):e233944. doi: 10.1001/jamanetworkopen.2023.3944 (PMC10031392; doi:10.1001/jamanetworkopen.2023.3944)
Supplement: Supplement 2. — Data Sharing Statement [file jamanetwopen-e233944-s002.pdf]

Data Sharing Statement

Ford. Associations Between Mother-Adolescent and Father-Adolescent Relationships and Young Adult Health. *JAMA Netw Open*. Published March 21, 2023. doi:10.1001/jamanetworkopen.2023.3944

Data
Data available: No

Additional Information
Explanation for why data not available: Data for this report were obtained via a user contract from the core Add Health Study. This data is available to others via a user contract from the core Add Health study team.
